# Radiology of fibrosis. Part I: Thoracic organs

**DOI:** 10.1186/s12967-024-05244-1

**Published:** 2024-07-02

**Authors:** Sofia Maria Tarchi, Mary Salvatore, Philip Lichtenstein, Thillai Sekar, Kathleen Capaccione, Lyndon Luk, Hiram Shaish, Jasnit Makkar, Elise Desperito, Jay Leb, Benjamin Navot, Jonathan Goldstein, Sherelle Laifer, Volkan Beylergil, Hong Ma, Sachin Jambawalikar, Dwight Aberle, Belinda D’Souza, Stuart Bentley-Hibbert, Monica Pernia Marin

**Affiliations:** 1 Department of Biomedical Sciences, Humanitas University, Milan, Italy; 2 Department of Radiology, Columbia University Irving Medical Center, 630 W 168th Street, New York, NY 10032 USA

**Keywords:** Fibrosis, Thoracic organs, Imaging

## Abstract

Sustained injury from factors such as hypoxia, infection, or physical damage may provoke improper tissue repair and the anomalous deposition of connective tissue that causes fibrosis. This phenomenon may take place in any organ, ultimately leading to their dysfunction and eventual failure. Tissue fibrosis has also been found to be central in both the process of carcinogenesis and cancer progression. Thus, its prompt diagnosis and regular monitoring is necessary for implementing effective disease-modifying interventions aiming to reduce mortality and improve overall quality of life. While significant research has been conducted on these subjects, a comprehensive understanding of how their relationship manifests through modern imaging techniques remains to be established. This work intends to provide a comprehensive overview of imaging technologies relevant to the detection of fibrosis affecting thoracic organs as well as to explore potential future advancements in this field.

**Supplementary Information:**

The online version contains supplementary material available at 10.1186/s12967-024-05244-1.

## Background

**Fig. 1:**
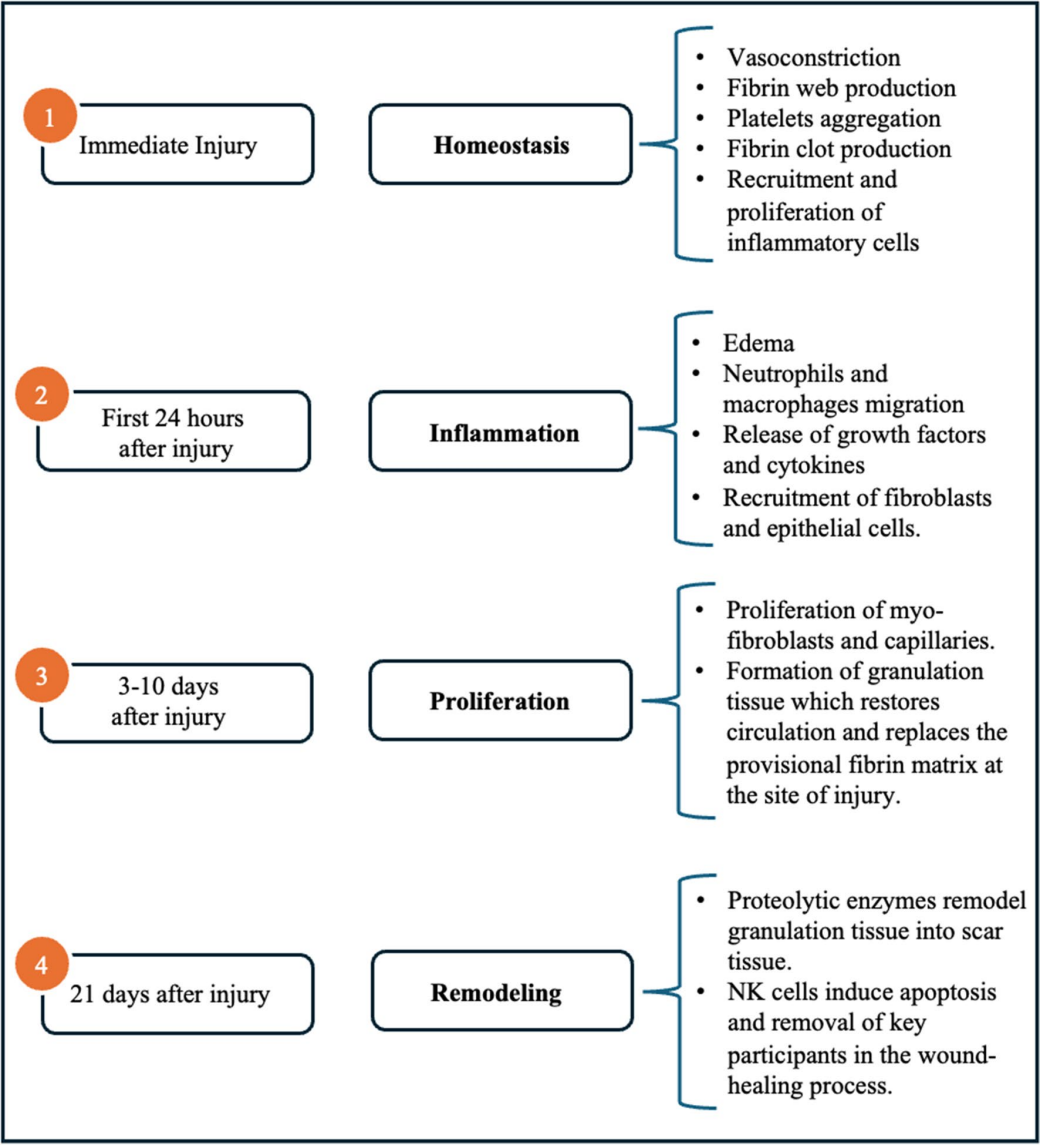
Diagram of the normal wound healing sequence. *NK* natural killer

**Fig. 2:**
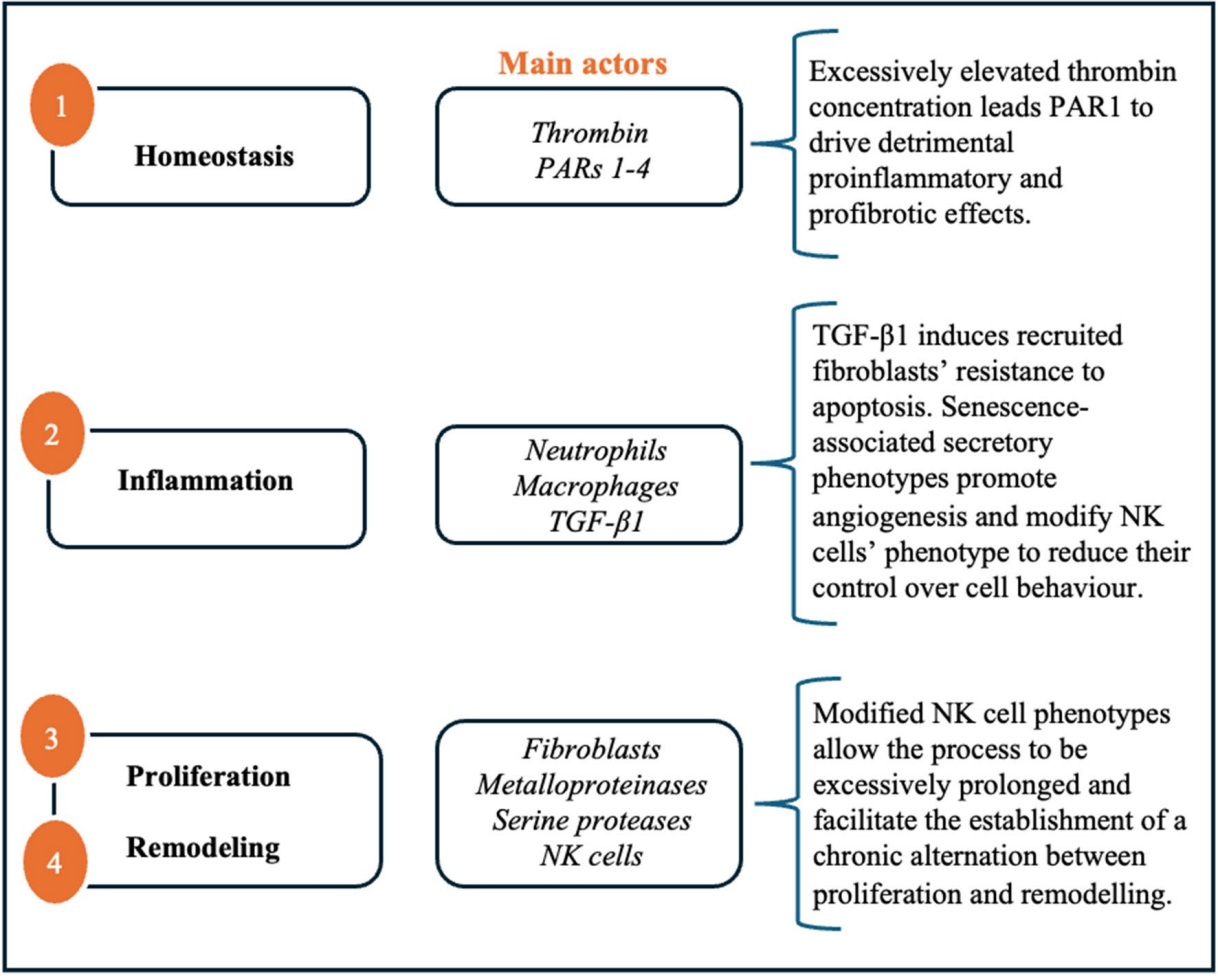
Diagram of the main pathological processes that occur in aberrant wound healing and lead to tissue fibrosis. *PARs* G protein-coupled Protease Activated Receptors. *TGF-β1* Transforming Groth Factor *β1*, *NK* natural killer

It's estimated that up to 45% of deaths in the industrialized world may be traced back to fibrosis, a pathological process resulting from complications in tissue repair and having the potential to structurally and functionally affect any organ through the excessive deposition of connective tissue [1–4]. Indeed, pathological response to tissue damage may determine an undue protraction of the physiological four-fold wound healing mechanism—typically comprising hemostasis, inflammation, proliferation, and remodeling—resulting in chronic inflammation, aberrant fibroblast proliferation, exaggerated collagen deposition, and a sequent imbalance in the alternation between scar formation and remodeling (Figs. 1, 2) [3, 5]. Today, chronic inflammation-related fibrosis is widely accepted to play a crucial role in initiation tumor development, with an estimated association with up to 20% of cancers [2]. Recognizing the potential implications this datum has on quality of life and general healthcare burden, there is a pressing need for a more in-depth knowledge of the interconnectedness of wound healing, chronic inflammation, and the ensuing fibrosis, to encourage subsequent research into cancer insurgence and prevention.

Hemostasis, characterized by vasoconstriction and the establishment of a temporary fibrin web within which active platelets become enmeshed, begins soon after injury [5–7]. This process is driven by thrombin, a pluripotent serine protease which catalyzes the proteolytic conversion of soluble plasma fibrinogen into an insoluble fibrin clot and recruits proliferating fibroblasts to the wound site [8, 9]. In physiological conditions, thrombin’s procoagulant activity is mediated by a family of four proteolytic receptors: G protein-coupled Protease Activated Receptors (PARs 1–4). However, compelling experimental evidence suggests that elevated coagulation protease concentrations lead PAR1 to drive detrimental proinflammatory and profibrotic effects instead [9, 10].

Inflammation intensifies 24 h after wounding when excessive exudation of fluid and plasma proteins from resident vessels causes the surrounding tissue to swell, the organ’s size to increase, and its density to decrease [3, 11]. Contemporaneously, neutrophils and macrophages migrate to the wound site, initiating the clearance of pathogens and matrix debris as well as releasing growth factors and cytokines, which play a crucial role in recruiting and activating fibroblasts and epithelial cells [5–7]. Kulasekaran et al. found that in aberrant wound healing transforming growth factor β1 (TGF-β1) induces recruited fibroblast’s resistance to apoptosis, allowing them to develop senescence-associated secretory phenotype with altered secretory profiles-including proinflammatory proteins, growth factors, and angiogenic factors—having the potential to influence the tissue microenvironment, promoting angiogenesis and modifying the phenotype of natural killer cells (NK cells), thereby diminishing their control over cellular behavior [2, 12–14].

Angiogenesis takes place 3–10 days after the injury occurs. Myo-fibroblasts and new capillaries proliferate to form granulation tissue which gradually restores circulation and replaces the provisional fibrin matrix at the site of injury [3, 6, 7].

During the fourth and final step in the wound healing process, metalloproteinases and serine proteases remodel granulation tissue into scar tissue whose tensile strength characteristics more adequately resemble those of healthy tissue [5–7]. Under physiological conditions, NK cells facilitate the apoptosis and removal of key participants in the healing process, leading it to gradually subside. Instead, in fibrosis, modified NK cell phenotypes allow the process to be excessively prolonged and facilitate the establishment of a chronic alternation between scar formation and remodelling [5].

The fact that the processes of wound healing and fibrogenesis appear so intricately interconnected, suggests a strong causal link between them. While significant research has been conducted on these subjects, a comprehensive understanding of how their relationship manifests through modern imaging techniques remains to be established. Recognizing the profound implications that advancements in this field may bring, in this work, we aim to outline how fibrosis manifests across various thoracic organs and provide a comprehensive overview of the key imaging technologies employed for its detection.

This is the first instalment of a three-part series focused on three thoracic organs of significance: the Breast, Lung, and Heart. Subsequent parts of this series are titled “Radiology of Fibrosis Part II: Abdominal Organs” and “Radiology of Fibrosis Part III: Urogenital Organs” will delve into discussions regarding abdominal and urogenital fibrosis, respectively. By structuring our work in this manner, we hope to have provided the readership with a clear image of a complex issue, paving the way for future betterment of clinical practice.

### Breast fibrosis

#### Mechanism of injury

The term benign breast disease (BBD) encompasses an array of non-malignant disorders commonly found via percutaneous biopsy in about 50% of females above the age of 30, although urbanization and socioeconomic background can result in marked variations [15, 16]. BBD is widely regarded to be the result of disordered proliferation of breast tissue resulting in a variety of abnormal developments, the most common of which are fibrocystic changes, mastalgia, and the formation of fibroadenomas [17, 18]. The term “fibrocystic changes” refers to a variety of clinical and histopathological modifications of the female mammary gland including cellular hyperplasia and the development of fluid filled cysts [17]. Mammographic imaging of BBD patients reveals a dense pattern of proliferation of glandular elements and supporting stroma, paired with the obliteration of mammary acini and ducts, as well as the decrease in adipose tissue proportions [16, 18, 19]. Alowami et al. found this density increase to primarily reflect alterations of the architecture and composition of stroma, the largest tissue fraction in breasts, as opposed to the glandular tissue, in which no difference in density was measured [16, 20]. Notably, increased mammographic density was also found to be linked to increased density of lumican and decorin, proteoglycans implicated in both stromal integrity and growth pathways due to their ability to bind TGF-β1 and its receptor, epidermal growth factor [20]. While the disorder’s progression has been shown to be influenced by age, menopausal status, and diet, its etiology is not yet known with certainty [20]. Even so, several hypotheses have been advanced including genetic predispositions, hormonal imbalances affecting both endogenous and exogenous sex steroids, and the upregulation of circulating growth factors [15, 19, 21]. It has been found that the strongest hormone-based association to increased mammographic density is increased levels of estrogen—physiologically dedicated to the regulation of ductal proliferation—and decreased levels of progesterone—physiologically dedicated to the regulation of ductal differentiation [15, 21]. Randomized control trials have demonstrated that exogenous administration of such hormones is related to statistically significant increases in breast density [21]. Furthermore, a clinical trial conducted by Brisson et al. found that long term (1–5 years) administration of tamoxifen, an estrogen receptor modulator, can reduce epithelial and stromal proliferation in breast tissue among premenopausal females, further supporting the hypothesis that breast density follows exposure to estrogens [21, 22].

With regards to the genetic influence on percent mammographic breast density, while no clear-cut links between polymorphisms in specific genes involved in steroid hormone metabolism and breast density have been drawn, significant evidence points to the presence of a genetic influence consistent with mendelian patterns of inheritance [23–26]. In a correlation study between monozygotic and dizygotic twins and the heritability of dense tissue on mammography carried out by Boyd et al., it was found that population variation was strongly influenced by genetic factors, with estimates ranging from 60 to 75%. Figures which were reduced by just 10 percent following adjustment for known major epidemiologic risk factors [27]. Interestingly, the correlation between monozygotic twins in percent mammographic density was approximately twice as strong as that between dizygotic twins—0.61 to 0.67 for monozygotic pairs and 0.25–0.27 for dizygotic pairs—a finding that is consistent with an additive genetic cause [27]. Finally, in premenopausal females, insulin-like growth factor I (IGF-I) and insulin-like growth factor-binding protein 3 (IGFBP-3) have been found to have, respectively, positive and negative correlation to increased mammographic density [21, 28]. This is unsurprising given IGF-I’s role in breast tissue proliferation, differentiation, and survival, and IGFBP-3’s role as an inhibitor of proliferation and promoter of mammary gland involution [21, 28].

Lastly, while it is true that some degree of BBD may occur in 50–60% of healthy females, particularly those in their middle and late reproductive periods, it is generally accepted that stromal hyperplasia is one of the strongest predictors of breast cancer risk [15, 21]. Females whose breasts contain at least 75% dense tissue are at a 4–6 times greater risk of breast cancer incidence than females with entirely fatty breasts [21]. Malik et al. found that 7% of imaging-based stromal hyperplasia diagnoses were later upgraded to malignancies on repeat biopsy, thus stressing the need for a more thorough understanding of the proliferative processes underlying BDD [29].

#### Mammography

The medical imaging technologies best accepted for breast density detection are digital mammography, US, and MRI, with mammography currently considered to be the gold standard [30, 31]. This low-dose x-ray system allows for the subjective yet effective classification of breast density into one of four severity categories defined by the Breast Imaging Reporting and Data System (BI-RADS) set forth by the American College of Radiology [32]. It is currently the only tool that has been shown to decrease breast cancer mortality rate by more than 30% through early detection [30, 31, 33, 34]. However, mammography only allows for 2D projections, causing physiological and pathologic breast tissue images to overlap [30, 35, 36]. A solution to this problem is offered by digital breast tomosynthesis (DBT), which consists of the stacked reconstruction of breast tissue images acquired in series, allowing for improved lesion detection, characterization, and localization [30, 35, 36]. Compared to simple mammography, DBT adjunct mammography is associated with increased cancer detection ranging from 1.2 to 4.6 per 1000 examinations, while also reducing false positive rates by 15% [35, 36]. DBT imaging is associated with increased acquisition and interpretation time [36]. Although mammography is currently considered to be the best screening tool available, it is not perfect, particularly in the case of dense breast parenchyma [33]. While 80–98% of masses are detected in those with fatty breast tissue, mammography’s sensitivity drops down to 30–48% in those with dense breast tissue [30, 33, 34]. An alarming datum since increased breast density has been proven to be an independent risk factor for breast cancer development [33]. For this reason, mammography is only considered to be the imaging modality of choice for females above 40–45 years of age, and not for the younger population whose breast density is, on average, higher [30, 33].

#### US

Ongoing research has demonstrated the value of breast US imaging adjunct mammography as a superior mass detection tool, especially in patients with increased breast density and mammographically occult findings [33, 34]. As a supplemental screening modality, US has been shown to allow for detailed characterization of most masses, even in the early stages [33–35, 37]. Furthermore, its sensitivity of 75% is significantly increased compared to that of independent mammograms, 64% [33]. Results from multiple studies have shown how, in dense-breasted patients, this combination determines an incremental mass detection rate of 2.3–4.6 for every 1000 people screened, in comparison to mammography alone [33–35]. In so doing, the addition of US screening promises to reduce morbidity and mortality while continuing to contain costs, be readily available, and well tolerated by patients [33–35, 37]. However, the operator dependent nature of this imaging modality paired with its lower specificity and positive predictive values limits its ability to be a stand-alone modality [34, 35]. One solution to these shortcomings is provided by the automated whole-breast US (ABUS), an adjunct to mammography which, in those with greater than 50% breast density, has resulted in the detection of 12.3 pertinent findings per 1000 patients, compared to 4.6 per 1000 by mammography alone [35].

#### CT

Breast computed tomography (bCT) is an emerging modality that provides high quality images and improves diagnostic accuracy over the current gold standard for the early detection and diagnosis of breast cancer [38–41]. It is a fully 3-dimensional tomographic technology in which 300–500 projections of the breast are acquired in a single circular scan allowing for a comprehensive reconstruction of the structure also making use of injected contrast media [38, 40, 42, 43]. bCT provides high quality images with no need for breast compression, removal of tissue overlaps, and with shorter acquisition time [39, 42]. Painful compression of the breast is spared, improving patient comfort, and potentially leading to increased screening compliance [39–41, 43]. Furthermore, the 3D nature of bCT has the potential to decrease false-negative exams in dense breast tissue where malignant lesions may be difficult to discern by removing the superimposition of benign and malignant breast tissue [39–44]. Studies have shown that detection rates for malignant masses are significantly improved at contrast-enhanced breast CT (CEbCT) than at mammography, tomosynthesis, or unenhanced breast CT [38, 39, 43, 45]. The sensitivity, specificity, and accuracy of chest CT for breast cancer detection have been reported to be 84.21%, 99.3%, and 98.68% compared to 78.95%, 93.78%, and 93.16% for mammography [44]. Malignant microcalcifications have been detected more clearly on CEbCT than on nonenhanced bCT but similarly on mammography [38, 39, 43, 45]. Instead, benign calcifications continue to be better visualized at mammography than at CEbCT [38, 43]. This imaging modality has been shown to be effective in discriminating malignant from benign lesions and microcalcifications [43]. This is possible because of the significantly greater contrast enhancement detected in cancerous tissue with respects to benign lesions [38, 43]. In so doing, CEbCT improves diagnostic specificity while lowering the number of false positives and halving the need for follow-up procedures [38, 42–44]. Finally, the primary drawbacks to diagnostic imaging through CT are the need for contrast enhancement with the potential for contrast reactions and the use of ionizing radiation [42, 43]. However, preliminary data has shown that the use of a radiation dose similar to that used in mammography nonetheless results in superior performance [42].

#### MRI

Breast MRI is one of today’s main methods for problem solving in the realm of breast disease diagnosis [46, 47]. In terms of sensitivity for the detection of invasive breast cancer, MRI outperforms the US adjunct mammography 75% to 32% [46, 48]. Combining MRI and mammography further increases sensitivity to 84% [48]. Furthermore, MRI significantly increased detection of early breast cancer beyond that seen with mammography or mammography combined with US [37]. These advantages over other breast imaging techniques have been shown to particularly benefit people at high risk of developing breast cancer, such as those with family history of the disease, those who are known to carry BRCA mutations, and their untested first-degree relatives [37, 46, 48, 49]. As such, the American Cancer Society recommends annual MRI based screening to those who fall into these categories [49]. Despite MRI’s very high true positive rate, it also presents with high false positive and false negative rates [35, 46]. Furthermore, it requires the injection of intravenous gadolinium and is expensive, found to be cost effective only when administered to those in the previously outlined high risk categories [35, 46, 48]. Finally, it is still debated whether MRI screening grants a clear survival advantage [48].

#### Nuclear medicine

While screening mammography rates are falling, an increasing number of low dose chest CTs, abdomino-pelvic MRIs, and 18-FDG-PET-CTs are being performed each year for diseases of the chest not related to the breast tissue itself [31, 50–53]. Multiple studies have shown this widespread use of imaging modalities to have led to an ever-increasing rate of incidental breast lesion discovery [31, 52, 53]. Indeed, it is common for significant amounts of breast tissue to be accidentally visible during such examinations, increasing the probability of breast findings in the field-of-view [50, 54]. Additionally, some portions of the breast such as the far medial aspect are difficult to visualize on mammography and might be better seen on CT, MRI, or 18-FDG-PET-CT [31]. For example, Bignotti et al. found that in 3.6% of their patients undergoing MRI investigation incidental breast densities were discovered, and 18.7% of these were later found to be clinically relevant [50]. The detection of clinically relevant incidental breast findings has been found to vary from 0.3% on CT, to 6% on 18-FDG-PET-CT [50]. Thus, chest radiologists should be aware of the benign and malignant appearances of breast parenchyma to facilitate diagnosis and treatment of incidental breast findings [31, 52, 53, 55]. Many are advocating for the inclusion of the entire breast tissue on screening and diagnostic scans obtained for other reasons, exposing the patient to no additional radiation, lost time, or expense, while contemporaneously increasing potential to diagnose breast lesions [31, 32, 52, 53, 56].

Finally, mammoscintigraphy, also known as breast-specific gamma imaging, is a noninvasive nuclear medicine diagnostic evaluation technique adjuvant to screening mammography [35, 57]. It involves the intravenous injection of Technetium 99-labeled sestamibi with subsequent imaging of the breast via SPECT gamma camera, which detects areas of increased radiotracer uptake [35, 57]. However, this technology is not currently in clinical use due to MRI’s increased prevalence and ability to provide similar information at higher quality [57].

#### Future directions

**Fig. 3:**
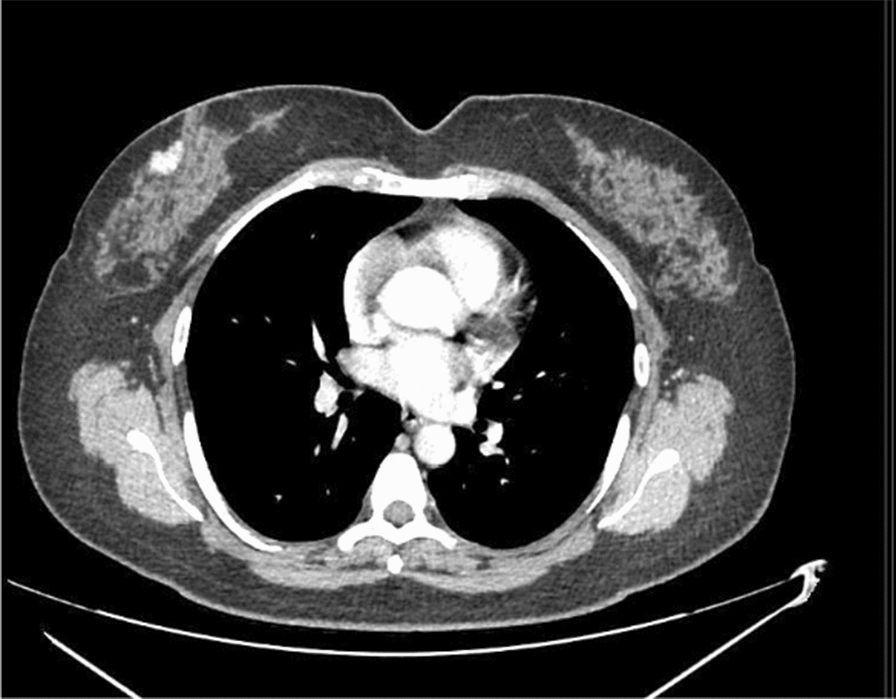
Thoracic CT (axial plane) showing the presence of right breast cancer

Benefits and drawbacks of each imaging technique discussed above are summarized in Table 1. Among the proposed alternatives, the authors of this review feel thoracic CT to be the most promising. When the entire breast is included in the soft tissue series, it can be visualized in mediastinal window settings and allow for early diagnosis of breast cancer, even before clinical presentation, and with limited radiation exposure [31, 51, 56]. The measurement of Hounsfield units has demonstrated to be the most important parameter to differentiate benign from malignant lesions in the breast [44]. In absence of additional tests or patient recall, chest CT correctly diagnosed breast cancer (p < 0.0001) compared to mammography [44]. These are promising results that could change the future of breast cancer screening guidelines (Fig. 3).

**Table 1:** Breast fibrosis imaging—pros/cons with respects to the gold standard

Breast fibrosis imaging		
US	PROs	Earlier detection, Increased sensitivity, Reduced morbidity, Reduced mortality, Low cost, Readily available, Well tolerated
	CONs	Operator dependent, Decreased specificity, Decreased positive predictive value
ABUS	PROs	Automated, Increased specificity, Increased positive predictive value, Improved performance in those with high breast density
	CONs	Limited supporting evidence
BCT^b^	PROs	High quality images, Increased accuracy, Earlier detection, Comprehensive reconstruction, Decreased acquisition time, Decreased false negatives, Improved field-of-view
	CONs	Use of ionizing radiation
CEBCT^b^	PROs	Improved malignant mass detection, Increased sensitivity, Increased specificity, Increased accuracy, Improved detection of malignant microcalcifications, Effective in differentiating between malignant/benign/calcific lesions, Decreased need for follow-ups, Improved field-of-view
	CONs	Decreased detection of benign microcalcifications, Need for contrast enhancement, Use of ionizing radiation
CE MRI	PROs	Increased sensitivity, Earlier detection, Benefits those at high risk of breast cancer, Increased true positive rate, Improved field-of-view
	CONs	High false positive rate, high false negative rate, Need for IV gadolinium, Expensive, Unclear survival advantage
18-FDG-PET-CT	PROs	Improved field-of-view
	CONs	Use of ionizing radiation, Long duration, Low availability, Expensive
Mammoscintigraphy	PROs	Structural and functional imaging
	CONs	Use of contrast enhancement, Not readily available
Mammography^a^	PROs	Low X-ray dose, Decreased Mortality, Limited field of view
	CONs	Subjective, 2D Projection, Lower sensitivity in case of dense breasts, Breast compression
DBT	PROs	Improved lesion detection/characterization/localization with respects to mammography, Increased cancer detection, Decreased false positive rates
	CONs	Increased acquisition time, Increased interpretation time

^a^Gold standard

^b^Promising future techniques

The authors of this review consider CEbCT to be another promising alternative. It is an emerging modality that provides high quality images and improves upon diagnostic sensitivity, specificity, and accuracy of the current gold standard [38, 41, 44]. It is a fully 3-dimensional tomographic technology in which 300–500 projections of the breast are acquired in a single circular scan allowing for a comprehensive reconstruction of the structure also making use of injected contrast media [38, 40, 42, 43]. bCT provides high quality images with no need for breast compression, removal of tissue overlaps, and with shorter acquisition time [39–44]. CEbCT has been shown to be effective in discriminating malignant from benign lesions and microcalcifications, thus improving diagnostic specificity while lowering the number of false positives and halving the need for follow-up bioptic procedures [38, 42–44]. While the primary drawback to diagnostic imaging through CT is the use of ionizing radiation, preliminary data has shown that the use of a radiation dose similar to that used in mammography nonetheless results in superior performance [42, 43].

### Lung fibrosis

#### Mechanism of injury

Idiopathic pulmonary fibrosis (IPF) is the most common form of idiopathic interstitial pneumonia, a heterogenous group of non-neoplastic diffuse parenchymal lung diseases characterized by stereotyped histological patterns [58, 59]. Initially they are inflammatory in nature and, if persistent, may result in parenchymal fibrosis and architectural remodelling [59]. IPF is a progressive and irreversible disorder causing gradual clinical deterioration, typically resulting in respiratory failure within 3 to 6 years of diagnosis [58, 60–62]. It has been found that, over the course of their illness, one in five patients will develop acute exacerbation of IPF (AE-IPF), a potentially life-threatening deterioration of respiratory function causing severe symptoms—most prominent of which is progressive dyspnea with associated cough, increased sputum, and fever—and a rapid decline in quality of life [62–66]. The disease has long been considered a chronic inflammatory process in response to severe epithelial injury as substantiated by histopathological evidence [58, 67, 68]. The original approach to IPF treatment was an anti-inflammatory three-drug regimen of immunosuppression (prednisone and azathioprine) in conjunction with N-acetylcysteine [69]. The role of inflammation in IPF was challenged in the 2012 PANTHER-IPF trial. In this randomized, double-blind, placebo-controlled study carried out by the Idiopathic Pulmonary Fibrosis Clinical Research Network, IPF patient rates of mortality, hospitalization, and other adverse events were found to increase in response to administration of the anti-inflammatory three-drug regimen, providing clinicians with compelling evidence against the involvement of inflammation in IPF’s physiopathology [69]. Despite the PANTER-IPF trial’s surprising results, many feel that anti-inflammatory medication may have a role in early disease. Bringardner et al. maintain that inflammation plays a distinct, albeit nontraditional, role in the pathogenesis of this disease. For example, they advance the direct inflammatory hypothesis, according to which, during AE-IPF, the bronchoalveolar (BAL) fluid is invaded by inflammatory agents, in particular by macrophage colony-stimulating factor (M-CSF) [70]. Murine models have demonstrated the pivotal role of M-CSF in regulating monocyte survival, proliferation, and differentiation. It has been found to directly stimulate the monocytes’ production of fibroblast recruitment factors CCL2 and CCL12 which play an important role in the advancement of pulmonary fibrosis, thus implying the centrality of inflammation in IPF’s progression [70]. Beyond the presence of M-CSF, the BAL of patients with IPF has been characterized by significantly elevated concentrations of monocyte chemotactic protein-1 (MCP-1) and interleukin-8 (IL-8) [67]. While IL-8 acts as a neutrophil chemotactic factor, bringing neutrophils from the peripheral circulation to the diseased lesion, MCP-1 causes an influx of monocytes into the pulmonary alveoli instead [67, 71]. Here, monocytes differentiate into fibrogenic macrophages, further increasing collagen production [67]. Thus, inflammation’s role in the furtherance of this fibrotic lung disorder is unmistakable, allowing for it to be classified as a chronic inflammatory process. The histopathological finding unique to IPF is termed spatial–temporal heterogeneity: a patchy alternation of normal and fibrotic tissue in various pathological stages of progression [58, 59]. The fibrotic destruction of the lung parenchyma is distributed in a subpleural, paraseptal, and lobular manner [59, 66]. Lobular distortion is greatest in the lower peripheral lobes which are heavier, nodular, and shrunken [59, 60]. The same histological arrangement is shared by microscopic honeycombing, the necessary precursor to macroscopic honeycombing, defined in the glossary of terms for thoracic imaging of the Fleischer Society as the complete destruction of lung architecture, presenting an array of cystic air spaces having similar dimensions and thick walls [72, 73]. Finally, scattered immature fibroblastic foci are commonly found at the interface between fibrotic and normal parenchyma.

#### CT

IPF diagnosis relies upon a combination of medical history taking, physical examination, laboratory findings, pulmonary function tests, imaging of the chest, and histopathology [74]. The 2018 international IPF diagnostic guidelines indicate high-resolution computed tomography (HRCT) to be the imaging gold standard, having been shown to be the most sensitive option [74–76]. Resultant images, obtained during full inspiration, reveal usual interstitial pneumonia pattern with or without superimposed diffuse alveolar damage, predominantly in the organizing phase [62, 77]. These often heterogeneous reticular abnormalities present subpleural and basal predominance with a progressive gradient toward the organ’s base [59, 75–77]. Ground-glass opacifications (GGO) leading to an increase in lung density given by the occupation of alveolar airspaces, can be found in areas of subpleural reticular density [62, 72, 74–76, 78]. Furthermore, honeycombing with or without peripheral traction bronchiectasis and the presence of irregular interlobular septal thickening are also common findings [74–77]. Even so, HRCT has been shown to be limited in its ability to detect disease progression and response to therapy [79]. Alternative imaging techniques, though less common and having varying diagnostic potentials, offer valid solutions to this problem [76]. Examples of these are Quantitative CT (Q-CT), gaseous, inhaled contrast agent enhanced magnetic resonance imaging (MRI), and 18 F-fluorodeoxyglucose (FDG) enhanced positron emission tomography (PET) [76, 79].

Emerging Q-CT methods have shown promise in more accurately assessing structural alterations, disease severity, and clinical evolution [76, 79]. This technology consists of a pattern recognition software which extracts specific pattern characteristics from traditional CT images [76, 79]. Extracted information is then processed via automated textural analysis machine learning algorithms, designed to detect IPF patterns that may have been otherwise overlooked [76]. In so doing, Q-CT provides a more rapid and reproducible alternative, decreasing inter-observer inconsistencies [76, 80, 81].

#### MRI

A radiation free alternative to conventional metrics is provided by gaseous, inhaled contrast agent enhanced MRI, a novel technique that has shown improved diagnostic and prognostic capacity [76, 79]. To date, most related research has focused on so-called hyperpolarized gases like helium (He) and xenon (Xe) [79]. Given that the distribution of such gasses mimics that of oxygen, inhaled contrast agent enhanced MRI allows for quantitative mapping of pulmonary functional patterns [79]. Indeed, Xe diffusion has been shown to be significantly elevated in regions of traction bronchiectasis and honeycombing, thus greatly facilitating IPF diagnosis [79]. Other significant developments in the evaluation of the lung parenchyma with MRI have been the utilization of a gradient echo sequence and a single-shot fast spin echo sequence with reduced echo time (TE) [82]. Examples of the latter are ultrashort echo time (UTE) MRI and zero echo time (ZTE) MRI. A gradient echo sequence allows for a better visualization of the tissue/blood density in the lung parenchyma while a shorter TE increases the MR signals helpful to recognize fine pulmonary structures that remained otherwise undetected with conventional methods [82–84]. The shorter the TE the greater the potential to evaluate lung parenchyma [82, 85].

#### Nuclear medicine

Finally, earlier identification of IPF may be made possible using FDG enhanced PET [76]. Indeed, the role of PET imaging in those with active sarcoidosis, a systemic granulomatous inflammation which evolves into fibrotic lung disease in 20% of all cases has been highlighted by Keijsers et al. [86, 87]. This imaging technique has been used to quantify lung fibrosis in patients with Scleroderma-related interstitial lung disease, a disorder characterized by systemic inflammation and progressive scarring of the lungs that leads to respiratory failure [88, 89]. The use of FDG allows for the discrimination of varying degrees of disease severity, serving as a valuable tool for mortality risk assessment, aiding prognosis, and treatment determination [76].

#### Future directions

**Fig. 4:**
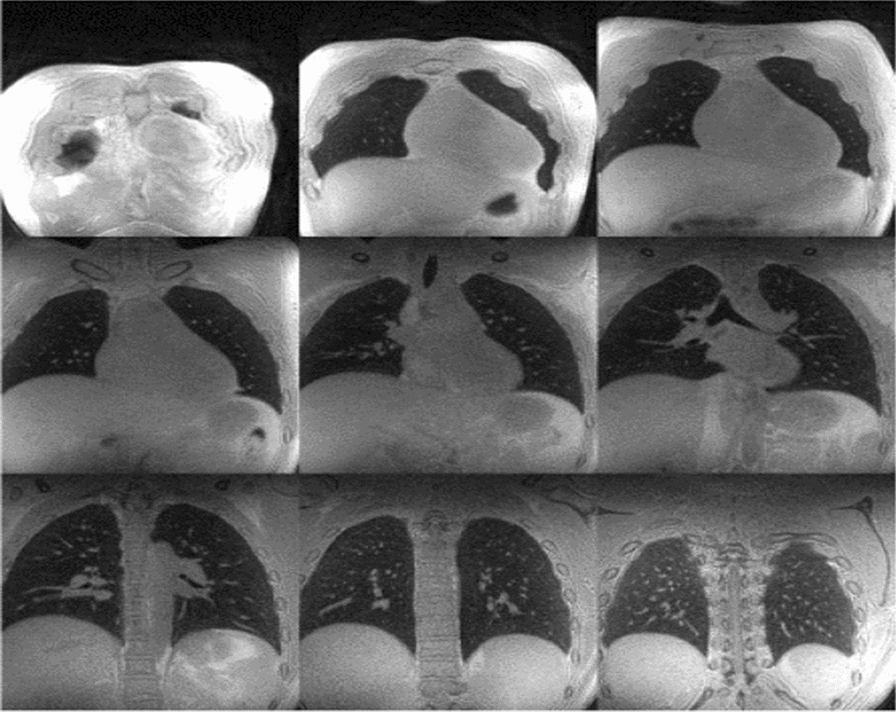
Coronal planes of UTE MRI and ZTE MRI of the lungs

Benefits and drawbacks of each imaging technique discussed above are summarized in Table 2. Among the proposed alternatives, the authors of this review feel UTE MRI and ZTE MRI to be the most promising. These methods are shorter and well tolerated while providing a highly detailed visualization of the lung parenchyma [82, 85]. Furthermore, Bae et al. has reported that the incorporation of high-resolution volumetric ZTE sequence to routine MRI is feasible [90]. With regards to image quality and small-nodule detection, ZTE MRI had better signal-to-noise ratio as well as contrast-to-noise ratio and demonstrated to be superior to UTE MRI (p < 0.05) [90] (Fig. 4).

**Table 2:** Lung fibrosis imaging—pros/cons with respects to the gold standard

Lung fibrosis imaging		
HRCT^a^	PROs	High sensitivity
	CONs	Limited ability to detect disease progression/response to therapy
Q-CT^b^	PROs	Improved ability to detect disease progression/response to therapy, Increased accuracy, Automated, Rapid, Reproducible, Decreased inter-observer variability, No contrast enhancement
	CONs	Not readily available
Xe/He diffusion MRI	PROs	Improved ability to detect disease progression/response to therapy, Increased accuracy, Automated, Rapid, Reproducible, Decreased inter-observer variability, No radiation
	CONs	Not readily available
18-FDG-PET-CT	PROs	Improved ability to detect disease progression/response to therapy, Early diagnosis, Improved discrimination of varying degrees of disease severity
	CONs	Use of ionizing radiation, Long duration, Low availability, Expensive
Gradient echo sequence MRI	PROs	Improved imaging of tissue/blood density
	CONs	Not readily available
UTE/ZTE MRI^b^	PROs	Improved imaging of fine pulmonary structures, Rapid, Well tolerated, Improved signal-to-noise ratio, Improved contrast-to-noise ratio
	CONs	Not readily available

^a^Gold standard

^b^Promising future techniques

The authors of this review consider Q-CT as another promising imaging tool for the assessment of lung fibrosis. This emerging technology provides a more rapid and reproducible alternative, decreasing inter-observer inconsistencies while more accurately assessing structural alterations, disease severity, and clinical evolution [76, 79–81]. Furthermore, this technology simplifies the imaging process by removing the need for contrast agents, making use, instead, of a pattern recognition software to extract specific pattern characteristics from traditional CT images [76, 79]. Extracted information is then processed via automated textural analysis machine learning algorithms, designed to detect IPF patterns that may have been otherwise overlooked [76].

## COVID-19 and lung fibrosis

During the COVID-19 pandemic, clinicians had the opportunity to witness the rapid evolution of lung inflammation into fibrosis to an extent that had not been previously possible. SARS-CoV-2 virus contagion has been found to affect alveolar epithelial cells much in the same way that AE-IPF has been reported to. The pathophysiology of this illness elucidates why respiratory symptoms—including cough, fever, and shortness of breath—are so common among the infected. The virus enters host cells via the angiotensin-converting enzyme 2 protein, which is abundant in both type II alveolar epithelial cells and vascular endothelial cells [91, 92]. Following infection of the host species, COVID-19 induces endothelial cell dysfunction (ECD), a systemic condition in which the endothelium loses its physiological properties and regulatory functions [91]. ECD induces a procoagulant state through the actions of thrombin, which activates platelets and converts fibrinogen into fibrin, thus promoting clot formation [93]. Furthermore, it augments inflammation through the activity of PAR-1 [94]. the negative feedback loops which tightly regulate its synthesis can also be compromised by inflammation itself. Consequently, an overactive cytokine response is established leading to a vicious cycle between inflammation and coagulation. The ensuing exacerbation of the inflammatory process predisposes lung parenchyma to the development of perfusion abnormalities [95]. It is possible to assess blood flow and extent of ischemia in the lung with the help of dual-energy contrast-enhanced lung CT through which they appear as peripheral lung opacities—given by decreased perfusion—surrounded by a halo of vascular enlargement—given by increased perfusion [96–98]. Damage to the basement membrane leads to increased alveolar capillary permeability with inflammatory cell infiltration and interstitial edema [97, 99, 100]. Excess fluid is detectable via HRCT in the form of increased lung density, GGO [78]. COVID-19 associated GGO presents a peripheral, subpleural, bilateral, and basal arrangement, similar to the distribution of IPF [78].

### Cardiac fibrosis

#### Mechanism of injury

Myocardial fibrosis consists of excessive expansion of the cardiac interstitium through deposition of ECM proteins, often leading to impaired electric conductance and reduced systolic and diastolic function [101–103]. Cardiac fibrosis has been found to be linked to many cardiovascular diseases including ischemic heart disease, hypertension, and heart failure [102, 104]. We can distinguish four types of cardiac fibrosis: replacement fibrosis, reactive fibrosis, infiltrative interstitial fibrosis, and endomyocardial fibrosis. In replacement fibrosis elevated levels of type I collagen and ECM replace necrotic cardiomyocytes following events that damage their membrane integrity [101, 102, 104]. In reactive fibrosis diffused collagen deposition determines increased interstitial compartment volume without myocyte hypertrophy in response to increased pressure or volume loads [101, 102, 104]. Infiltrative interstitial fibrosis is seen in conditions such as amyloidosis or Anderson-Fabry disease [101, 102, 104]. Endomyocardial fibrosis is one of the primary causes of pediatric congestive heart failure affecting the apical ventricular endocardium in children under the age of 2 years in tropical and subtropical regions [101, 102, 104].

Cardiac fibrosis is made up of three distinct phases: the initiating phase, the effective phase and the amplificative phase [102, 104, 105]. During initiation, cardiac insult induces an increase in the levels of circulating and myocardial pro-fibrotic growth factors and cytokines synthesized by fibroblasts [102, 104, 105]^.^ During the effective phase, these pro-fibrotic growth factors and cytokines activate an extensive range of complexly interconnected pro-fibrotic molecular routes aimed at obtaining the trans-differentiation of fibroblasts into myofibroblasts [102, 104–106]. In so doing, the cells develop altered response to mechanical stress, upregulation of α-smooth muscle actin expression, and altered synthesis of matrix metalloproteinases (MMPs) and tissue inhibitors of metalloproteinases (TIMPs) which regulate the dynamic balance between ECM deposition and degradation [102, 104]. During amplification, these same factors act on fibroblasts themselves, forming a positive feedback loop which amplifies fibrotic signals eventually leading to the development of cardiac fibrosis [102, 104, 105].

Among the above mentioned pro-fibrotic molecular routes are the renin–angiotensin–aldosterone system (RAAS), fibrogenic growth factor driven pathways, and those induced by pro-inflammatory cytokines [102, 104, 106]. According to the RAAS, macrophages and fibroblasts infiltrating the injured heart produce renin and angiotensin converting enzyme [102, 104]. This is followed by the generation of angiotensin II, which stimulates cardiac fibroblast proliferation and enhances their collagen-synthetic activity through AT1 receptor-dependent interactions and through the mediatory effects of reactive oxygen species (ROS) [102, 104]. Among the fibrogenic growth factor pathways we find those determined by platelet-derived growth factor (PDGF) and by TGF-β1 [102, 104]^.^ Following cardiac injury, latent TGF-β1 is activated by ROS and there is overexpression of PDGF, leading to fibroblast proliferation and trans-differentiation, as well as increased ECM synthesis via downstream intracellular pathways [102, 104, 106]. Among the inflammatory signals implicated in the generation of cardiac fibrosis, several in vitro studies have found multifunctional cytokines TNF- α and IL-6 to be crucial in its development [106]. Several chemotactic cytokines known as chemokines, particularly MCP-1, have been found to mediate reactive fibrotic effects through chemotactic recruitment of pro-fibrotic mononuclear cells and fibroblast progenitors [102, 104]. MCPs have been found to enhance fibroblast proliferation and myofibroblast differentiation, thus upregulating collagen synthesis and TGF-β1 expression [102, 104]. Because of their involvement in the development of myocardial fibrosis, RAAS and TGF-β1 are common therapeutic targets [101]. For example, Direct Renin Inhibitors target RAAS’ rate limiting step by binding directly to renin, thus attenuating its pro-fibrotic effects [107]. Instead, Pirfenidone and Tranilast have been found to suppress TGF-β1 transcription and its downstream effects, thus downregulating fibroblast collagen production [107]. Despite evidence supporting the effectiveness of these drugs, none have yet been approved for clinical use against cardiac fibrosis. Therefore, more research is warranted to explore alternative treatments [107].

#### US

In the fibrotic heart, myocardial tissue velocity and deformation parameters may be reduced due to excess collagen deposition [108]. Thus, a possible noninvasive measure of myocardial collagen deposition is the basic 2D echocardiographic imaging technique, a highly available, reproducible, and inexpensive alternative [108, 109]. This technology serves to identify increased endocardial thickening and consequent myocardial stiffness, a characteristic of diffuse fibrosis, through the quantification of myocardial strain and strain rate deformation parameters [108, 109]. Indeed, impaired myocardial velocity and resultant cardiac dysfunction have been found to be symptomatic of interstitial scarring [108]. A pertinent example of this technology in action is offered by a Weidemann et al. study in which real-time 2D colour Doppler myocardial imaging data was used to derive strain rate profiles, subsequently integrated over time to derive the strain rate and strain curves [110]. In the presence of fibrosis, it was found that the strain curves consistently and reliably presented a typical deformation pattern consisting of two clearly identifiable peaks: the double peak sign, the first and second peak, respectively corresponding to peak systolic and peak post-systolic strain rate [110]. Thus, it may be said that doppler echocardiographic imaging can be used for the assessment of the presence of regional fibrosis in an easy, reproducible, accurate, and highly sensitive manner [110]. Even so, 2D echocardiography presents with sensitivity and specificity values which are too low for comprehensive tissue characterization [109]. A valid noninvasive US-based imaging alternative to this technology is offered by speckle tracking echocardiography (STE) [109, 111]. This technique is proven to indirectly evaluate myocardial dysfunction through the semiautomated analysis of deformation parameters in 3 spatial directions [109, 111]. To do so, STE tracks the movement in space of speckles—spots generated by the interaction between the US beam and myocardial fibers—on routine 2D echography throughout the cardiac cycle [111]. It too presents with advantageous high availability, high repeatability, and non-invasivity [109, 111].

#### MRI

The major imaging technique employed to assess cardiac fibrosis is cardiac magnetic resonance (CMR) which can be of two types: late gadolinium enhancement (LGE) technique and T1 mapping technique [101]. LGE allows detection and quantification of replacement fibrosis with high sensitivity and specificity [101, 112]. Within fibrotic tissue, gadolinium concentration is increased due to decreased capillary density, causing T_1_ shortening and resulting in bright signal intensity in the CMR image [112]. T1 mapping technique improves upon LGE tissue characterization by enabling direct quantification of the degree of fibrosis via a standardized scale. Heightened spatial resolution is achieved via the use of 1.5-T magnetic resonance imaging scanners within a single breath hold [112–114].

The most commonly employed noninvasive techniques to assess myocardial fibrosis include CMR, PET, and tissue doppler echocardiography [101, 108, 112]. Ideally, accurate noninvasive tools should allow for accurate detection and prognostication of fibrosis-based cardiac dysfunction, enabling early deployment of antifibrotic therapy without the risks associated with invasive methods [108, 114].

CMR imaging fulfills these criteria and is thus used extensively for the comprehensive soft-tissue characterization of myocardial mass, volume, function, and perfusion in routine clinical settings [101, 108, 112–114]. Its widespread availability paired with its unequaled accuracy and reproducibility have rendered CMR the gold standard technique with which to collect data regarding cardiac replacement fibrosis [101, 108, 112, 113]. In CMR images, signal intensity is determined by the time it takes hydrogen nuclei protons immersed in a static magnetic field to relax [112, 114]. This process may be characterized using three main relaxation parameters—T1, longitudinal relaxation time, T2, the ideal transverse relaxation time, and T2*, the effective transverse relaxation time—which values vary from one tissue type to another, as well as from one physiopathological state to another (inflammation, edema, fibrosis, etc.) within the same tissue type [112]. T1 mapping is a promising new approach for evaluation of cardiac fibrosis. Fibrotic myocardium retains contrast within the extracellular space and shortens T1 with enhancement of fibrosis relative to normal myocardial tissue. The enhancement is irregular and in a typical distribution [115]. Although CMR is considered to be the reference standard in this area of study having high spatial resolution and specificity, its routine use is limited by its high costs and time-consuming image acquisition [109, 111].

In the context of cardiac fibrosis, the LGE CMR approach is one of the most established techniques [101]. The intravenous administration of LGE allows for the identification of areas of discrete myocardial replacement fibrosis with high sensitivity and specificity [101, 112]. Within diffusely fibrotic myocardium, decreased capillary density coupled with increased ECM volume determines prolonged washout time and greater retention of the contrast agent, leading to longitudinal relaxation time (T_1_) shortening and voxel signal hyperenhancement with respects to healthy myocardium [108, 112–114, 116]. Kim et al. found that the extent of abnormally high intensity regions may be used to infer true size and severity of microvascular damage [116]. LGE CMR imaging technology’s ability to identify scar tissue depends on its ability to compare adjacent regions’ signal intensities [114]. Thus, while it has become the gold standard technique for imaging focal myocardial fibrosis, in the context of diffuse fibrosis these differences in signal intensity are lacking, limiting the technology’s ability to quantify scar tissue [113, 114]. A solution to this problem is offered by T1 mapping which allows for intrinsic inversion time (T1) quantification of each myocardial voxel within the evaluated tissue [112–114]. Conversely, in T_2_ mapping, transverse relaxation time has been found to be prolonged rather than shortened in regions of edema, inflammation, and fibrosis [113]. Finally, quantification of T2*, the transverse relaxation time in the presence of static magnetic field irregularities, enables quantitative tissue characterization as well as the detection of changes in myocardial oxygenation via the exploitation of the difference in magnetic state between oxyhemoglobin and deoxyhemoglobin, as the former increases while the latter decreases T_2_* time [113]. An alternative CMR application which has been gaining popularity in recent times is feature-tracking CMR which allows for the detection and quantification of decreased myocardial deformation, a common functional consequence of diffuse myocardial fibrosis, even before symptoms arise [109].

CMR is generally contraindicated in patients with claustrophobia, renal dysfunction, pacemakers or implantable cardioverter-defibrillators. Thus, detecting myocardial scaring in these patients would require the adoption of a safer alternative [117, 118]. One such suitable approach could be contrast enhanced multi-detector CT (ceMDCT) [117–119]. Emerging data suggests that this technology can be used in conjunction with simple ECG and CMR to provide both anatomical and functional tissue characterization while still being well tolerated by patients and having excellent spatial and temporal resolution [109, 120]. Much like in CMR, ceMDCT makes use of an extravascular, extracellular contrast agent to assess myocardial extracellular volume (ECV), a marker of myocardial tissue remodelling [109, 118–121]. Indeed, the employed iodinated radiographic contrast agents become trapped within ECM-rich regions of the myocardium much in the same way that gadolinium becomes trapped in the context of LGE MRI [117]. Myocardial ECV derived via ceMDCT has been shown to be comparable to that derived via CMR imaging [118–120]. Finally, several studies have demonstrated ceMDCT’s ability to accurately quantify the extent of myocardial scarring with a good correspondence to both histological findings and the gold standard, CMR [109, 117, 119]. Even so, ceMDCT’s interobserver reproducibility and contrast resolution were found to be significantly lower than those of CMR thus rendering additional radiation exposure unjustifiable [109, 119, 120, 122].

### Nuclear medicine

Another valid alternative to CMR is offered by PET currently considered to be the gold standard technique for noninvasive quantitative measurements of myocardial perfusion [108, 113, 123]. It is a nuclear imaging technique which employs either oxygen-15-labeled water or nitrogen-13-labeled ammonia to monitor coronary perfusion, or 18 F‐fluorodeoxyglucose to monitor myocardial metabolism [101, 123]. A mismatch between the detected coronary perfusion and metabolism may be indicative of replacement fibrosis [101]. Furthermore, via PET, it is possible to calculate the perfusable tissue index, an indirect marker of fibrosis obtained through the subtraction of the amount of nonperfusable tissue from its perfusable counterpart [108]. However, PET presents with higher costs and lower availability with respects to its competitor, CMR [101, 113, 123]. Its usage exposes patients to ionizing radiation and requires an on‐site cyclotron with which to obtain tracers [101, 113, 123].

#### Future directions

**Fig. 5:**
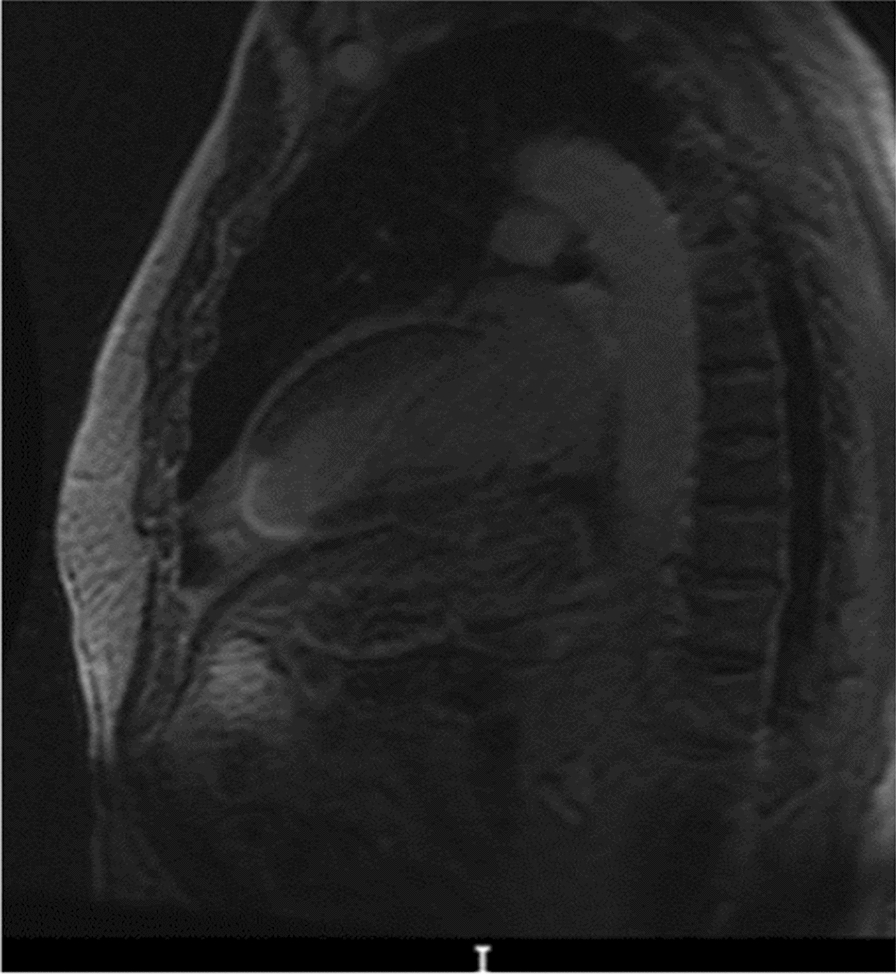
MRI (sagittal plane) showing cardiac fibrosis

Benefits and drawbacks of each imaging technique discussed above are summarized in Table 3. Among the proposed alternatives, the authors of this review feel cardiac MRI (Fig. 5) and PET to be the most promising. PET is currently considered to be the gold standard technique for noninvasive quantitative measurements of myocardial perfusion [108, 113, 123]. It is a nuclear imaging technique which employs either oxygen-15-labeled water or nitrogen-13-labeled ammonia to monitor coronary perfusion, and 18 F‐fluorodeoxyglucose to monitor myocardial metabolism [101, 123]. A mismatch between the detected coronary perfusion and metabolism may be indicative of replacement fibrosis [101]. Furthermore, it allows for the calculation of the perfusable tissue index, an indirect marker of fibrosis [108]. Instead, LGE CMR, with its high sensitivity, specificity, and accuracy, stands out for its effectiveness in early detection and reproducibility, coupled with increased spatial resolution and specificity. Similarly, CMR utilizing the T1 Mapping Technique offers direct fibrosis quantification on a standardized scale, alongside increased spatial resolution, high accuracy, and specificity, enabling early detection with reproducibility and improved signal-to-noise ratio.

**Table 3:** Heart fibrosis imaging—pros/cons with respects to the gold standard

Heart fibrosis imaging		
2D Echocardiography	PROs	Readily available, Reproducible, Inexpensive
	CONs	Low sensitivity, Low specificity
Feature tracking CMR	PROs	Early detection
	CONs	Contraindicated in patients with claustrophobia/renal dysfunction/pacemaker/implantable cardioverter-defibrillator
STE	PROs	Semiautomated, Readily available, Reproducible
	CONs	Not readily available
CEMDCT	PROs	Suitable for patients with claustrophobia/renal dysfunction/pacemaker/implantable cardioverter-defibrillator, Both anatomical and functional imaging, Increased spatial resolution, Increased temporal resolution, High accuracy
	CONs	Need for contrast enhancement, Decreased inter-observer reproducibility, Decreased contrast resolution, Radiation
LGE CMR^b^	PROs	High sensitivity, High specificity, High accuracy, Early detection, Readily available, Reproducible, Increased spatial resolution, High specificity
	CONs	Need for contrast enhancement, Expensive, Time consuming, Low signal-to-noise ratio
CMR—T1 mapping technique	PROs	Direct quantification of fibrosis via standardized scale, Increased spatial resolution, High accuracy, Early detection, Readily available, Reproducible, High specificity, Inside signal-to-noise ratio
	CONs	Expensive, Time consuming
PET^a,b^	PROs	Functional imaging
	CONs	Expensive, Low availability, Use of ionizing radiation, Requires on-site cyclotron

^a^Gold standard

^b^Promising future techniques

## Conclusions

Fibrosis is a pathological process resulting from complications in tissue repair and having the potential to structurally and functionally affect any organ through the excessive deposition of connective tissue [1–4]. Indeed, pathological response to tissue damage may determine an undue protraction of the physiological four-fold wound healing mechanism—typically comprising hemostasis, inflammation, proliferation, and remodeling—resulting in chronic inflammation, aberrant fibroblast proliferation, exaggerated collagen deposition, and a sequent imbalance in the alternation between scar formation and remodeling [3, 5]. While significant research has been conducted on these subjects, a comprehensive understanding of how their relationship manifests through modern imaging techniques remains to be established. Recognizing the profound implications that advancements in this field may bring, in this work, we aimed to outline how fibrosis manifests across various thoracic organs and provide a comprehensive overview of the key imaging technologies employed for its detection. Our review of all pertinent literature highlights that US, CT, MR and PET are the most commonly adopted imaging technologies for fibrosis detection across the considered organs. Exceptions include only highly tissue-specific techniques like digital mammography for breast fibrosis. Overall, we believe that, among the proposed imaging technique alternatives, MRI emerges as the most promising due to its heightened soft tissue contrast and absence of ionizing radiation. Moreover, MRI's widespread availability, ability for full-body scanning, and reported lower incidence of allergic reactions compared to other contrast-exploiting techniques like X-ray and CT enhance its appeal in clinical settings (Table 4).

**Table 4:** Authors’ opinion regarding the most promising radiology techniques to diagnose fibrosis in each organ

Suspected affected organ	Promising radiology techniques for diagnosis
Breast	Chest CT and CEbCT
Lungs	UTE/ZTE MRI and Q-CT
Heart	Cardiac MRI and PET

### Supplementary Information

Supplementary Material 1

## Data Availability

Data sharing not applicable to this article as no datasets were generated or analyzed during the current study.

## Abbreviations

FDG: 18 F-Fluorodeoxyglucose
AE-IPF: Acute exacerbation of IPF
ABUS: Automated whole-breast us
BBD: Benign breast disease
BCT: Breast computed tomography
BI-RADS: Breast imaging reporting and data system
BAL: Bronchoalveolar
CMR: Cardiac magnetic resonance
CEMDCT: Contrast enhanced multi-detector CT
CEBCT: Contrast-enhanced breast CT
DBT: Digital breast tomosynthesis
TE: Echo time
ECV: Extracellular volume
He: Helium
HRCT: High resolution computed tomography
IPF: Idiopathic pulmonary fibrosis
IGF-I: Insulin-like growth factor I
IGFBP-3: Insulin-like growth factor-binding protein 3
IL-8: Interleukin-8
LGE: Late gadolinium enhancement
M-CSF: Macrophage colony-stimulating factor
MRI: Magnetic resonance imaging
MMPS: Matrix metalloproteinases
MCP-1: Monocyte chemotactic protein-1
NK Cells: Natural killer cells
PDGF: Platelet-derived growth factor
PET: Positron emission tomography
PAR: Protease activated receptors
Q-CT: Quantitative CT
ROS: Reactive oxygen species
RAAS: Renin–angiotensin–aldosterone system
STE: Speckle tracking echocardiography
TIMPS: Tissue inhibitors of metalloproteinases
TGF-Β1: Transforming growth factor Β1
US: Ultrasound
UTE: Ultrashort echo time
Xe: Xenon
ZTE: Zero echo time
